# Barriers and enablers to source plasma donation by gay, bisexual and other men who have sex with men under revised eligibility criteria: protocol for a multiple stakeholder feasibility study

**DOI:** 10.1186/s12961-020-00643-4

**Published:** 2020-11-02

**Authors:** Elisabeth Vesnaver, Mindy Goldman, Sheila O’Brien, Paul MacPherson, Terrie Butler-Foster, Don Lapierre, Joanne Otis, Dana V. Devine, Marc Germain, Andrew Rosser, Richard MacDonagh, Taylor Randall, William Osbourne-Sorrell, Broderic Clement-Thorne, Taim Bilal Al-Bakri, Kyle A. Rubini, Nolan E. Hill, Justin Presseau

**Affiliations:** 1 School of Epidemiology and Public Health, University of Ottawa, Ottawa, Canada; 2 Medical Affairs and Innovation, Canadian Blood Services, Ottawa, Canada; 3 Medicine, The Ottawa Hospital, Ottawa, Canada; 4 Medical Affairs and Innovation, Canadian Blood Services, London, Canada; 5 Department of Sexology, Université du Québec À Montréal, Montreal, Canada; 6 Canadian Blood Services, Vancouver, Canada; 7 Héma-Québec, Medical Affairs, Quebec City, Canada; 8 PRIDE London President, London, Canada; 9 Local Advisory Group, London, Canada; 10 Centre for Sexuality, Calgary, Canada; 11 Clinical Epidemiology Program, Ottawa Hospital Research Institute, Ottawa, Canada; 12 Department of Biochemistry, Microbiology and Immunology, University of Ottawa, Ottawa, Canada; 13 Centre for Blood Research, University of British Columbia, Vancouver, Canada

**Keywords:** gbMSM, Plasma donation, Integrated knowledge translation, Co-production, Behaviour change, Blood donation eligibility

## Abstract

**Background:**

Blood donation policy in Canada for gay, bisexual and other men who have had sex with men (gbMSM) has changed progressively in the last decade from indefinite deferral to 3-month deferral from last male-to-male sex. Driven by safety data and overseen by the national regulator, more inclusive policies continue to redress the disparity in donation for gbMSM. At the same time, the need for source plasma to prepare fractionated blood products is growing worldwide. The collection and processing of source plasma ensures greater safety compared to whole blood donation with respect to transfusion-transmitted infection. This greater safety offers an opportunity to evolve policies for gbMSM from time-based to behaviour-based deferral using revised eligibility criteria. However, changing policies does not in itself necessarily guarantee that gbMSM will donate or that staff in donor clinics are ready to support them to do so. In anticipation of a move to behaviour-based donation screening for gbMSM in Canada, we aim to assess the acceptability of and perceived barriers and enablers to source plasma donation using revised screening criteria for gbMSM among key stakeholders to inform policy implementation strategies.

**Methods:**

This mixed-methods feasibility study will involve gbMSM and donor centre staff to understand modifiable barriers to implementing more inclusive eligibility criteria. Key informant interviews and surveys will be rooted in the Theoretical Domains Framework to identify modifiable factors associated with source plasma donation motives in gbMSM and training needs in donation centre staff. We will use an integrated knowledge translation approach involving a partnership between researchers, the national blood operator and gbMSM, situating knowledge users as key research team members to ensure their perspectives inform all aspects of the research.

**Discussion:**

Our integrated knowledge translation approach will provide a more comprehensive and collaborative understanding of blood operator and gbMSM needs while accelerating the implementation of study findings. Given the historical backdrop of the decades of exclusion of sexually active gbMSM from blood donation, this study has the potential not only to inform a process and policy for gbMSM to donate source plasma, a blood product, but also offers opportunities for new relationships between these knowledge users.

## Background

The demand for plasma proteins (e.g. immunoglobulins) continues to rise globally, outstripping the supply. Canada does not collect enough source plasma to meet the needs of its citizens and would benefit from widening the range of possible donors, especially to those who may be interested but limited from doing so, such as gay, bisexual and other men who have sex with men (gbMSM). Source plasma is a type of plasma donation that is frozen and then sent to a manufacturer for the production of specific plasma protein products such as intravenous immunoglobulins. The additional processing involves pathogen reduction and additional assurance of supply safety compared to whole blood donation with respect to transfusion-transmitted infection. This greater safety offers an opportunity to widen the range of possible source plasma donors with more inclusive eligibility screening.

### Evolving policy

In the early 1980s, in Canada and many other countries, blood donation by gbMSM was banned based on the higher seroprevalence of HIV in this group and because there was no test available to detect HIV infection at the time [1]. Donor criteria have moved progressively from a permanent deferral (cannot donate) to a 3-month deferral since the last time of sexual encounter with a goal of moving to behavioural risk screening for gbMSM donors. The risk of HIV infection is not the same for all gbMSM; behavioural risk screening would enable identification based on sexual behaviour rather than time since sexual encounter [2, 3]. Currently, Canadian blood donor criteria do not permit any sexually active gbMSM to donate whole blood or source plasma—men who have had sex with a man in the past 3 months are deferred. However, Canadian Blood Services, one of Canada’s two national blood operators, is reviewing its policies regarding source plasma donation in an effort to be more inclusive while maintaining the safety of the blood supply, including some gbMSM that are at low to no risk of HIV infection such as those in monogamous relationships.

It is likely that many in the general population, including gbMSM, are not yet as aware of what source plasma donation entails and how it differs from whole blood donation. Source plasma donation involves drawing whole blood, separating out the plasma, and then returning red and white blood cells and platelets back to the donor. The source plasma donation process takes approximately 45 min and can be donated as frequently as every week. Once collected, source plasma can be frozen and stored for months and the manufacture of plasma protein products includes several pathogen reduction steps to kill any residual infectious agents. Conversely, fresh components collected during whole blood donation, such as platelets and red blood cells, have a short storage period of up to 7 or 42 days, respectively, and are currently not subjected to the same pathogen reduction processes as source plasma in Canada. Both the longer storage time of source plasma before pooling and the pathogen reduction manufacturing processes involved in the production of plasma protein products make it feasible to apply additional safety steps that may allow some sexually active gbMSM to donate source plasma. Expanding the eligibility to gbMSM for source plasma donation first provides an opportunity to collect data on gbMSM donors whilst maximising the continued safety of the supply. Data on gbMSM donors is critical to support changes to whole blood screening policies; with current deferrals in place and gbMSM unable to donate, no such data can be collected.

### What would the revised eligibility criteria involve?

As of 2019, all male donors are asked ‘In the last 3 months, have you had sex with another man?’ Responding yes to this question results in a 3-month deferral from last sexual contact, which amounts to an indefinite deferral for a donor in an ongoing relationship. Revised eligibility criteria could include adding additional behavioural screening questions to those who answer ‘yes’ to identify gbMSM that are at low to no risk of HIV infection. The additional behavioural questions under consideration include:

1. Have you had sex with a new partner in the last 3 months?
2. Have you had sex with more than one partner in the last 3 months?
3. Are you in a mutually exclusive (monogamous) relationship?

These questions are based on those in use in jurisdictions such as France (quarantined plasma programme), Italy and Spain [4–6] as well as data generated in the ongoing Canadian research studies on gbMSM and blood donation. The use of the draft questions in this study will permit further validation of their clarity and acceptability, which may lead to modifications in the actual questions submitted to Health Canada for implementation in a source plasma collection site.

### A change in criteria is necessary but insufficient

Making donation possible is necessary but likely insufficient for a successful gbMSM plasma donation programme. Given the historical exclusion of gbMSM from any blood or blood product donation, any gbMSM donation programme would require general acceptability among gbMSM. In Canada, Caruso et al. [7] explored the acceptability among gbMSM of a plasma donation programme involving quarantining plasma until donors return 2 months or more after initial plasma donation and are retested for transmissible diseases, thus ensuring the safety of the supply prior to release. The programme was considered by some participants to reinforce the exclusion and discrimination experienced with whole blood deferral policies because gbMSM donors and their plasma are treated differently.

While behavioural risk screening is an approach that has previously been supported by gbMSM with respect to whole blood donation [8, 9], there is also a need to explore how gbMSM understand and would respond to the screening questions. Studies of gbMSM that have donated blood despite not being eligible reported that participants donated because they were uninformed about the policies or they assessed their own HIV risk to be low with a mixed understanding of the ‘window period’, the period in which new HIV infection is not detectable but potentially infectious [8–10]. Exploring how gbMSM understand the behavioural screening questions prior to implementation can help uncover and address the challenges of adherence to the policies.

### Balancing supply safety with equality and justice

The ineligibility of sexually active gbMSM from blood and plasma donation has attracted debate worldwide with regards to justice, exclusion, safety, risks and community [11–14]. Time-based deferrals that involve policies specific to gbMSM have been described by some as discriminatory, disproportionate to risk and unnecessarily strain the supply of available blood (and plasma) products [15, 16]. The gbMSM blood donor debate has ignited many gbMSM and allies to equate blood donation with equality and full citizenship and has resulted in lawsuits, boycotts of blood drives and petitions [16, 17]. The effects of group exclusion from blood donation can be long lasting as has been observed in the Haitian-Canadian community [18]. An extension of existing donation promotion strategies without such considerations is likely to miss the mark. Understanding how gbMSM view the historical context and changes of the policies in relation to how they view the proposed behavioural risk screening approach will generate insights for the development of appropriate strategies for communication of the policy and promotion of source plasma by gbMSM.

### Staff perspectives

The staff in donation clinics who would be tasked with implementing the revised eligibility criteria for gbMSM play a central role in the safety and success of such a programme. Alteration of the eligibility criteria may require additional training and staff may have views and perspectives that, if surfaced early, would help to ensure a feasible and acceptable roll-out of a gbMSM source plasma donation programme. Indeed, Hughes et al. explored the views of blood collection organisation staff on the transition from indefinite deferral to 12-month deferral in gbMSM in California [19]. They showed that, while some staff voiced reservations, most staff supported this transition. They also showed that staff valued their professionalism and would follow the regulations imposed by the Food and Drug Administration in the United States regardless of their own personal views. Furthermore, most staff expressed a desire for further training and materials. Findings such as these highlight the importance of understanding the potential barriers and enablers to the implementation of revised eligibility criteria to ensure that such a change could be feasibly implemented. However, the Hughes et al. study focused on whole blood donation and the move from indefinite to 12-month donation deferral in gbMSM. There is a need to explore such views in more detail in a Canadian setting for source plasma donation and for revised eligibility criteria that moves away from a time-since-sexual-encounter criterion.

### Understanding the challenges before jumping to solutions

While this is a promising opportunity for more inclusive donation to enhance the donor pool with a previously excluded segment of the population, it is important to assess the feasibility with key knowledge users prior to implementation. The proposed study is designed to fill this gap and provide information from gbMSM and donor centre staff on whether revised eligibility criteria for gbMSM is viewed to be feasible and acceptable and what factors could be adapted to enhance these features [20].

The implementation of revised eligibility criteria and gbMSM source plasma donation can be described as an intervention designed to support behaviour change in gbMSM. Due to the number of different knowledge users that may each require different targeted strategies, the intervention can be described as a ‘complex’ intervention. The United Kingdom Medical Research Council guidance on the development and evaluation of complex interventions highlights the importance of careful intervention development using appropriate theory and feasibility assessment prior to evaluation and implementation [21]. Although there are extant theories to draw from to understand first time blood donation (as it would be for most gbMSM), the application of behavioural science to source plasma donation is scant and, in Canada, source plasma donors are typically recruited from among whole blood donors [22]. Furthermore, the historical backdrop of exclusion of gbMSM from donation may have resulted in drivers that are unique to this population.

A systematic approach to intervention development can provide transparency and foster cumulative evidence to ensure that the strategies devised to support gbMSM in donating and staff in screening are fit for purpose. French et al. [23] proposed a four-step approach for designing theory-based implementation interventions that suggests key steps in the systematic development of an intervention to change behaviour as follows: first, identify who needs to do what differently; second, identify what barriers and enablers might be relevant; third, select change strategies and techniques that are fit for purpose to address identified barriers and enablers; and fourth, identify how change can best be measured. This study will focus on completing step two using a comprehensive theoretical framework.

To ensure the findings are useful to all knowledge users, this study will be rooted in an integrated knowledge translation (IKT) approach, a form of research co-production [24]. Research co-production is an equitable collaborative approach to research that meaningfully engages knowledge users who are directly impacted by the results of the research [25]. IKT is distinguished from other co-production approaches with its emphasis on collaboration with knowledge users that are in positions of power to create change [26], the focus of study is identified by knowledge users, and research focuses on “*generating real-life solutions to complex problems*” [27].

## Methods

### Aims and objectives

The purpose of this study is to assess the feasibility and acceptability of source plasma donation with revised eligibility criteria for gbMSM in two Canadian cities. Our specific objectives are to identify the following:

1. Views and experiences of gbMSM regarding source plasma donation, current eligibility criteria and revised eligibility criteria.
2. The acceptability of additional behavioural questions during the screening process from the perspective of gbMSM.
3. Potential barriers and enablers to source plasma donation from the perspective of gbMSM.
4. The acceptability of additional behavioural questions during the screening process from the perspective of donor centre staff.
5. Potential barriers and enablers from the perspective of donor centre staff in the donation clinic to implementing new eligibility criteria for gbMSM to donate and to inform the adaptations needed to centre flow and processes prior to piloting.
6. Consistencies and discrepancies between the two Canadian cities and implications for tailoring strategies to support gbMSM source plasma donation in each context.

### An integrated knowledge approach

Consistent with an IKT approach, the initial topic was born out of Canadian Blood Services seeking to better understand the feasibly of a source plasma programme for gbMSM with more inclusive donation eligibility criteria and the broader voice of gbMSM demanding greater equality as it relates to donation. The research questions were collaboratively developed by the research team consisting of scientists and collaborators from research institutions, Canada’s two national blood operators, and a local LGBT2Q+ community organisation to ensure diverse perspectives and expertise. While our team offers both insider and outsider perspectives with respect to the donor centre working environment and identifying as gbMSM, we sought out greater inclusion of gbMSM voices in the design and conduct of the study design. We first consulted with community members through outreach and engagement activities and these activities resulted in the formation of a Local Advisory Group of gbMSM. Local advisors are key members of the research team (rather than participants) who provide ongoing input in monthly group meetings and in individual exchanges by email or phone as needed. They provide feedback on study design, contribute to survey and interview guide development, and help to develop and facilitate the recruitment strategies. They will review and provide feedback of summaries of analysis and results, advise on next steps, and help disseminate findings.

### Study design

This mixed-methods feasibility study will explore the views of gbMSM and donor centre staff regarding source plasma donation and eligibility criteria to better understand the modifiable barriers and enablers to implementing revised eligibility criteria. We will use qualitative interviews and an online anonymous survey to identify the barriers and enablers to source plasma donation that may emerge with the implementation of revised screening criteria for source plasma donation by gbMSM. Qualitative interviews will be used to elicit potential barriers and enablers to implementing revised eligibility from the perspectives of donor centre staff.

#### Setting

This study will be conducted in London (Ontario) and Calgary (Alberta). Canadian Blood Services operate two dedicated source plasma donation centres that are located in London (Ontario) and Calgary (Alberta). These centres are potential locations for a first implementation of a gbMSM source plasma donation programme if approved by Health Canada. The study was first developed in London (Ontario) due to operational feasibility and strong relationships with the local gbMSM community. The research team sought additional funding to expand the project to Calgary (Alberta).

#### Analytic framework

Many factors may emerge as barriers and enablers to source plasma donation by gbMSM or to donor centre staff’s implementation of revised eligibility criteria. Solutions and strategies for encouraging and supporting donation should be tailored to address these barriers to ensure that the supports developed are fit for purpose [28]. Theories of behaviour provide a useful set of factors to consider when investigating barriers and enablers. By providing an understanding of which modifiable factors may be associated to implementing revised criteria, such theories provide a source of factors that could then be directly targeted to develop strategies and materials to support donation. There are many different theories that could be used as a basis for identifying such factors in a systematic way. A group of researchers sought to synthesise key content across 33 predominant theories and the 128 constructs within them. They developed the Theoretical Domains Framework (TDF) [29, 30], which summarises key factors from theory that are known to be associated with behaviour and behaviour change and is well suited to explore the full breadth of factors that are relevant in this behaviour and population. The TDF identifies 14 different modifiable factors, as follows: knowledge, skills, beliefs about capabilities, optimism, beliefs about consequences, intention, goals, professional/social role and identity, social influences, reinforcement, behavioural regulation, emotion, memory/attention/decision processes, and environmental context and resources. Clear guidance has been developed to use the TDF for developing qualitative interview guides [31] and quantitative surveys [32]. The TDF has been used broadly as a basis for understanding barriers and enablers in the healthcare setting and with the public [32–35]. Once identified, this approach specifically suggests particular strategies and behaviour change techniques that best suit addressing the barriers and enablers identified based on expert review and the evidence base [36].

### gbMSM perspectives

#### Qualitative interviews

##### Participants and recruitment

We will use a combination of purposive and snowball sampling to recruit adult (18+) gbMSM in London (Ontario) and Calgary (Alberta) as well as in surrounding communities for interviews. Purposive sampling will be used to recruit gbMSM who represent a breadth of age, cultural backgrounds and geographic locations (rural/urban). We will work with our local advisory groups, local organisations and social groups that provide services to gbMSM to help identify potential participants. In 2020, many countries, including Canada, were practicing social distancing to reduce the spread of COVID-19. In light of this, we will advertise on social media platforms with assistance from organisations that provide services to gbMSM. We recognise that this method of sampling may not reach those who are not active on social media and, as such, we will supplement this strategy with snowball sampling methods by inviting participants to recommend others for participation. Snowball sampling is well suited for the recruitment of traditionally underserved groups and those who experience stigmatisation, including gbMSM.

##### Procedure

We will conduct up to two semi-structured interviews per participant, by phone, scheduled over a period of 2–6 weeks. Informed consent will be obtained prior to the interview. Interviews will be approximately 60 min in length and audio-recorded for verbatim transcription. The use of a multiple interview format will promote the development of rapport over time, facilitating the discussion of sensitive and personal topics while allowing time for reflection and elaboration [37]. We will offer the option of one longer interview if participants prefer, to take a participant-centred approach to data collection. Field notes will be captured after each interview to assist with analysis and reflection on the impact of the interviewer’s positionality on the data generated. Interview participants will receive a CAD$ 20 gift card at each interview session to thank them for their time, to a maximum of CAD$ 40 per person. Participants that opt for one longer interview will receive a CAD$ 40 gift card.

##### Interview guide development

The first interview will explore the context of how source plasma donation is perceived by gbMSM using a semi-structured approach to interviewing. Key interview questions will be used to help define the areas to be explored but the interview style will remain flexible to allow for the discovery and discussion of topics of importance to the participant [38, 39] that may not have otherwise been thought of as pertinent by the research team [40]. The topic guide includes questions regarding experiences of donation, deferral or exclusion, views on current gbMSM donor deferral criteria, and the acceptability of the three behavioural screening questions suggested for inclusion in revised eligibility criteria.

The second interview will build on the first to elaborate on emerging themes and explore participants’ views regarding the implementation of revised eligibility criteria, potential impacts of revised eligibility criteria on donation practices, and possible barriers and enablers to source plasma donation. The topic guide for the second interview draws on existing literature [31] and previously developed guides [33] to assess if and how the identified barriers align with TDF domains. Interview guides will be reviewed by the study’s local advisory groups in each city and piloted prior to broad enrolment. At the end of the interviews, participants will be asked about the interview experience and this feedback will be considered and incorporated as appropriate.

##### Sample size

Our sample size will be determined by the available number of interviewees. We aim to recruit 15–20 men from each region to complete a series of two interviews. This sample size estimate is informed by the scope of the study, the nature of the topic and the use of a multiple interview format [41]. Given that data collection and analysis are concurrent, informational and thematic redundancy [42, 43] will be assessed on an ongoing basis and the number of interviews will largely be driven by the quality and richness of data [44]. Drawing on the literature, saturation is often reached within 15–20 TDF interviews [33, 34].

##### Data analysis

Interviews will be audio recorded, transcribed verbatim and de-identified prior to analysis. As an additional step, we will send each participant their written transcript to review for completeness and resonance. We will use NVivo12 to facilitate analysis. Data collection and analysis will be concurrent to generate emerging understanding of the research questions, which will inform both the sampling and the questions being asked [39]. We will conduct two phases of qualitative descriptive analysis—inductive thematic analysis [45] to identify, analyse and report patterns within the data, and theory-driven, directed content analysis to code barriers and enablers expressed by gbMSM to specific TDF domains [46]. Throughout, reflexive journaling will be used to capture the analytic process and any developing insights about the patterns in the data. Data from each region will be analysed separately as each context may have unique cultural, societal and political forces that shape the views of gbMSM regarding source plasma donation. We will contrast findings between cities to inform city-specific modifications for implementation.

During thematic analysis, we will follow the six analytic steps proposed by Braun and Clark [45]: becoming familiar with the data, generating initial codes, searching for themes, reviewing themes, defining and naming themes, and producing the report. We will then re-visit the data during directed content analysis and use guidance from the literature [31] to code barriers and enablers to source plasma donation expressed by gbMSM to specific TDF domains [46]. We will develop a code book to enhance the reliability of coding. To enhance the trustworthiness of our findings, we will use a combination of duplicate coding by two researchers trained in both inductive analysis and the use of the TDF, peer debriefing activities and consensus-building measures.

#### Online survey

##### Participants

We will invite adult gbMSM (aged 18+) living in London (Ontario) and Calgary (Alberta) to complete an anonymous questionnaire regarding their views about current and potential future barriers and enablers to donating source plasma. Questionnaire items will enable the assessment of respondents’ eligibility to donate according to the revised criteria but will not exclude participants to the survey on the basis of these criteria.

##### Recruitment and procedure

Our local advisory groups of gbMSM will facilitate recruitment by providing access to the venues and organisations through which a link to the online survey can be circulated as well as by advising on additional non-traditional venues for recruiting participants. We will offer the opportunity to enter a draw for one of ten pre-paid Visa gift cards valued at CAD$ 125 each.

##### Questionnaire development

The questionnaire will be designed for completion by adult gbMSM and will include screening questions to ensure that we involve our targeted respondents. We will use items from a TDF questionnaire previously assessed for its discriminant content validity [32] to assess barriers and enablers to donating source plasma if they were eligible under revised eligibility criteria. Appropriate language and response options will be informed by the interviews; thus, certain questions may be unique to each city. The questionnaire will be piloted by gbMSM prior to launching.

##### Data analysis

Our analytical approach is modelled on an approach used by Presseau et al. [33]. We will conduct descriptive analyses to identify mean scores and standard deviations on each of the 14 TDF domains. As donation represents a hypothetical behaviour for respondents at the moment, we will assess intention to donate as a proxy for actual donation (consistent with other donation studies) [47]. We will assess bi-variate associations between intention to donate source plasma with socio-demographic factors, including self-identified gender, eligibility to donate (based on revised criteria) and responses to each TDF domain. Informed by behavioural theory [33, 48], we will then conduct multiple regression analysis to investigate which variables are associated with gbMSM’s intention to donate source plasma. We will investigate whether these associations are moderated by their eligibility as determined by the revised criteria. If the surveys end up very similar between sites, we will analyse the combined sample and investigate whether the associations differ by location. We are powered to conduct independent analyses in each region if needed.

##### Sample size

For analysis of our survey data, for a regression model comprised of data covering 20 independent variables [i.e. 13 TDF domains (not including intention), as well age, self-identified gender, rural/urban, sex with new partner in last 12 months (yes/no), sex with more than one partner in last 3 months (yes/no), in a monogamous relationship (yes/no), married (yes/no)], we will require a total sample size of 314 participants (157 participants in each region) to detect a medium effect size (R^2^ = 0.15). We will aim to continue to recruit until this sample is exceeded or the recruitment period is completed (6 months).

### Staff perspectives

#### Participants and recruitment

We will interview English-speaking donor centre staff involved in applying current eligibility criteria or discussing eligibility with potential donors at the London donor centre. Recruitment will be facilitated by our stakeholder contacts at the donor centre. Nurses and donor care associates will be invited to participate in a one-on-one semi-structured telephone interview lasting 30–45 min. Interviews will focus on the barriers and enablers to using each of the proposed revised screening criteria.

Our approach to interview guide development, data analysis and sample size is rooted in the TDF framework and will follow the methods outlined for the interviews with gbMSM.

### Dissemination

The findings of the study may be of interest to diverse audiences and require a multi-pronged dissemination strategy. A key advantage of an IKT approach is that stakeholders are members of the research team and provide real-time guidance on the type of dissemination that is needed at different stages of the project. Our first priority is to work with our stakeholders to ensure they have the findings in an appropriate format and to develop a dissemination plan for their respective communities. We will collaborate with our local advisory groups of gbMSM to disseminate among their local communities and beyond, which may involve a website and online events or articles in gbMSM-focused venues. We will also work with our blood operator stakeholders to disseminate appropriately through their organisations and international networks. Beyond our stakeholders, dissemination of this work is likely relevant to stakeholders outside the traditional scientific audience and, thus, we will aim to publish in open-access journals or repositories and share these via social media to reach a broader audience.

### Limitations

Source plasma donation by sexually active gbMSM is not currently permitted. The data generated in this study is based on a hypothetical change in policy. The barriers and enablers will be elicited by asking respondents to answer to a hypothetical scenario, which may be different from their responses to real-life scenarios [49]. If revised eligibility is approved, ongoing investigation is needed alongside any initial implementation.

### Study status

This protocol is based on a grant funded by Health Canada, administered by Canadian Blood Services, and peer-reviewed by experts external to the funder and blood operator. The study has been reviewed and received ethics approval from the Ottawa Health Science Network Research Ethics Board (ID # 20190287-01H and 20200255-01H). The Canadian Blood Services Ethics Board has also reviewed and approved the parts of the study involving donor centre staff (ID 2019.020). At the time of submission, recruitment and data collection had begun with donor centre staff and gbMSM.

## Discussion

The findings from this study will contribute to our understanding of the views of gbMSM regarding source plasma donation and source plasma donation eligibility policies as well as of the perceived barriers and enablers to donating source plasma should they become eligible. Findings will help to inform the development of implementation strategies and support donation promotion if policies change to enable some sexually active gbMSM to donate. This study will also generate insights regarding staff anticipated barriers and enablers to implementing revised criteria for source plasma donation that will inform intervention development in preparation for wide-scale implementation if policy changes are approved by Health Canada.

A strength of using the TDF is that the domains have been mapped to specific evidence-based behaviour change techniques [36]. Thus, the findings will specify theory-driven and evidence-based strategies to be incorporated into future intervention development to address the barriers and enablers identified, increasing the likelihood that resulting strategies are likely to be effective. Although the findings are based on two collection sites, by rooting our approach in behaviour change theory, we will enhance the transferability of findings to other sites.

The multiple sources and types of data that will be collected in this study will enable a broader understanding of the needs to be addressed prior to implementation. Qualitative methods will help us to explore the full range of factors that may impact implementation by staff and donation among gbMSM. Quantitative findings will inform which of these factors identified by gbMSM are likely to have the greatest impact on donation intention, elucidating the areas most amenable to the targeted strategies to support donation.

Canada is not alone in its consideration of behaviour-risk screening for blood donation [50–52]. While many countries have followed a similar evolution to blood donation policies for gbMSM, the context in which these policies are made and enacted vary greatly. The contextual findings can help decision-makers and researchers in other jurisdictions to better situate the findings and assess the transferability of our findings to their context [42].

As of 2020, the public discourse related to blood donation by gbMSM was intensifying in response to the repeated calls for blood donation by blood operators internationally due to COVID-19-related blood shortages [53, 54]. There was also increasing public interest in plasma donation and the ineligibility of gbMSM due to the clinical trials investigating convalescent plasma as a treatment for COVID-19 [55, 56]. Convalescent plasma is a type of plasma collected from patients recovered from COVID-19; gbMSM who have recovered from COVID-19 are not eligible to donate. The iterative and adaptable nature of an IKT approach that engages both the blood operator and gbMSM combined with the flexibility of qualitative methods makes this project uniquely situated to adapt and respond to the shifting social context of the study.

Due to the decades of exclusion from blood and blood-product donation that gbMSM have experienced and the social and political movement that has arisen in response, there may be divisions between blood operators in different countries and gbMSM that will need to be bridged for gbMSM to engage enthusiastically with a source plasma donation programme. Research using IKT has the potential to create impact beyond addressing the research objectives. There is potential for the different knowledge user groups involved in the research to experience mutually beneficial learning cycles throughout the project’s stages of design, data collection, analysis, reporting and dissemination [25]. As each unique perspective contributes to the work, all knowledge users and researchers learn from these perspectives and may shift their own perspectives. Furthermore, in this process, there is the potential to strengthen relationships across knowledge users. Partnerships between gbMSM and Canadian blood operators can only aid in improving the development of appropriate and sensitive implementation supports and ultimately facilitate bringing gbMSM into the donor base.

## Conclusions

This research will explore the perceptions and experiences of gbMSM and donor centre staff regarding the feasibility and acceptability of implementing revised eligibility criteria for source plasma donation by gbMSM. The findings from this study will help refine or provide feedback on three proposed behaviour-based screening questions for donation eligibility and provide a basis for developing sensitive materials that could support a change in policy.

## Data Availability

Not applicable.

## Abbreviations

gbMSM: Gay, bisexual, and other men who have sex with men
IKT: Integrated knowledge translation
TDF: Theoretical Domains Framework
